# Inflammatory monocytes regulate Th1 oriented immunity to CpG adjuvanted protein vaccines through production of IL-12

**DOI:** 10.1038/s41598-017-06236-6

**Published:** 2017-07-20

**Authors:** S. De Koker, L. Van Hoecke, A. De Beuckelaer, K. Roose, K. Deswarte, M. A. Willart, P. Bogaert, T. Naessens, B. G. De Geest, X. Saelens, B. N. Lambrecht, J. Grooten

**Affiliations:** 1 Department of Biomedical Molecular Biology, Ghent University, Ghent, Belgium; 2 VIB Inflammation Research Center, Ghent University, Zwijnaarde, Belgium; 3 Department of Biochemistry, Ghent University, Ghent, Belgium; 4 VIB Medical Biotechnology Center, Ghent University, Ghent, Belgium; 5 Department of Respiratory Medicine, University Hospital Ghent, Ghent, Belgium; 6 Department of Pharmaceutics, Ghent University, Ghent, Belgium

## Abstract

Due to their capacity to skew T cell responses towards Th1 oriented immunity, oligonucleotides containing unmethylated CpG motifs (CpG) have emerged as interesting adjuvants for vaccination. Whereas the signalling pathways in response to CpG mediated TLR9 activation have been extensively documented at the level of the individual cell, little is however known on the precise identity of the innate immune cells that govern T cell priming and polarisation to CpG adjuvanted protein antigens *in vivo*. In this study, we demonstrate that optimal induction of Th1 oriented immunity to CpG adjuvanted protein vaccines requires the coordinated actions of conventional DCs and of monocytes. Whilst conventional DCs were required for antigen presentation and initial T cell priming, monocytes constitute the main source of the Th1 polarising cytokine IL-12.

## Introduction

Agonists of Toll-like receptors (TLRs) that recognize viral and bacterial nucleic acids (TLR3, 7, 8 and 9) have the capacity to skew T cell responses towards Th1 oriented immunity and have emerged as promising adjuvant candidates^1–3^. TLR9 is triggered by unmethylated cytosine-phosphate-guanine (CpG) oligodeoxynucleotide motifs present in viral and bacterial DNA^4, 5^. Interaction of CpG with TLR9 induces a signalling cascade that results in the recruitment of MAP kinases and the activation of transcription factors (NF-κB, AP1 and IRF-7)^6, 7^. Whereas the signalling pathways in response to TLR9 activation have been extensively documented at the level of the individual cell, little is however known on the precise identity of the innate immune cells that govern T cell priming and polarisation to CpG adjuvanted protein antigens *in vivo*.

Initial activation of effector T cell responses critically depends on three signals: antigen presentation to the T cell receptor (signal 1), co-stimulation (signal 2) and cytokine receptor signalling at the T cell surface (signal 3). Whilst signal 1 and signal 2 are sufficient to drive initial T cell proliferation, antigen-experienced T cells require signal 3 cytokines to acquire effector function and to differentiate into long lived memory T cells^8–10^. Signal 1 and signal 2 are typically provided by dendritic cells (DCs), the immune system’s most potent antigen presenting cells endowed with the unique capacity to prime naïve T cells. DCs are however highly heterogeneous and diverse subsets have been identified based on life cycle, ontogeny and function. Dependent on their route of entry into lymph nodes, DCs have been divided in lymph node resident DCs – which enter the lymph node directly from the bloodstream - and in migratory DCs - which reach the lymph nodes from peripheral tissues via the afferent lymphatics. The exact role of lymph node resident versus migratory DCs in priming T cell responses remains controversial and appears to be highly dependent on the nature of the antigen and of the inflammatory insult^11–18^. With regards to ontogeny, most DCs in steady state derive from a dedicated DC progenitor in the bone marrow. These DCs have been named conventional DCs and rely on Fms-like tyrosine kinase receptor 3 ligand (Flt3L) for their development^19–27^. Under inflammatory conditions however, also monocytes can give rise to a cell type reminiscent of a DC, which hence has been named an inflammatory or monocyte derived DC. This determination is largely based on their expression of CD11c and MHCII, the two cardinal markers used to identify DCs. Yet, to which extent monocyte derived DCs in fact also functionally resemble conventional DCs remains a matter of intensive debate^28–35^. Opposing findings regarding the capacity of monocyte derived DCs to act as antigen presenting cells have been reported^13, 34, 36–39^. Moreover, conclusions often have been confounded by the past lack of markers that reliably separate monocyte derived DCs from CD11b+ conventional DCs. Ambiguity also exists regarding the nature of the cells that deliver the necessary signal 3 to drive effector T cell differentiation^40^. In the most conservative model, signal 3 cytokines are provided by the same DC that also presents the antigen to the T cell. Nevertheless, signal 3 cytokines might also be supplied in *trans* by other DCs or even other innate immune cells that do not present the antigen themselves. Within this context, a growing number of studies have pinpointed monocytes as complex regulators of adaptive immunity. Monocytes are amongst the most abundant innate cells recruited to virtually any infectious insult and display a tremendous plasticity in secreting cytokines tailored towards the type of insult encountered^32, 38, 41–48^. The profound recruitment of monocytes to sites of inflammation thereby adds an additional, poorly understood layer to the complex cross-talk between pathogen/adjuvant, antigen presentation and inflammation that together shape the ensuing T cell response.

Intradermal injection of CpG has been reported to mobilize vast numbers of monocytes to the lymph nodes of mice^49^ and non-human primates^50^. In this study, we aimed to decipher whether and how monocytes regulate T cell effector responses to CpG adjuvanted protein vaccines. Through a set of *ex vivo* and *in vivo* antigen presentation assays, we identified migratory DCs as the main antigen presenting cells and initiators of T cell proliferation. Although migratory DCs comprised both conventional DCs and monocyte derived DCs, antigen presentation predominantly resided within the conventional DC population. Nevertheless, through secretion of vast amounts of IL-12, monocytes created the appropriate inflammatory environment that supported differentiation of antigen experienced T cells into Th1 T cells. Taken together, our findings reveal that optimal induction of effector T cell responses to CpG adjuvanted vaccines requires the coordinated actions of both conventional DCs and monocytes.

## Results

### CpG injection dramatically expands Ly6C^hi^ monocytes and DCs in the draining lymph nodes

**Figure 1:**
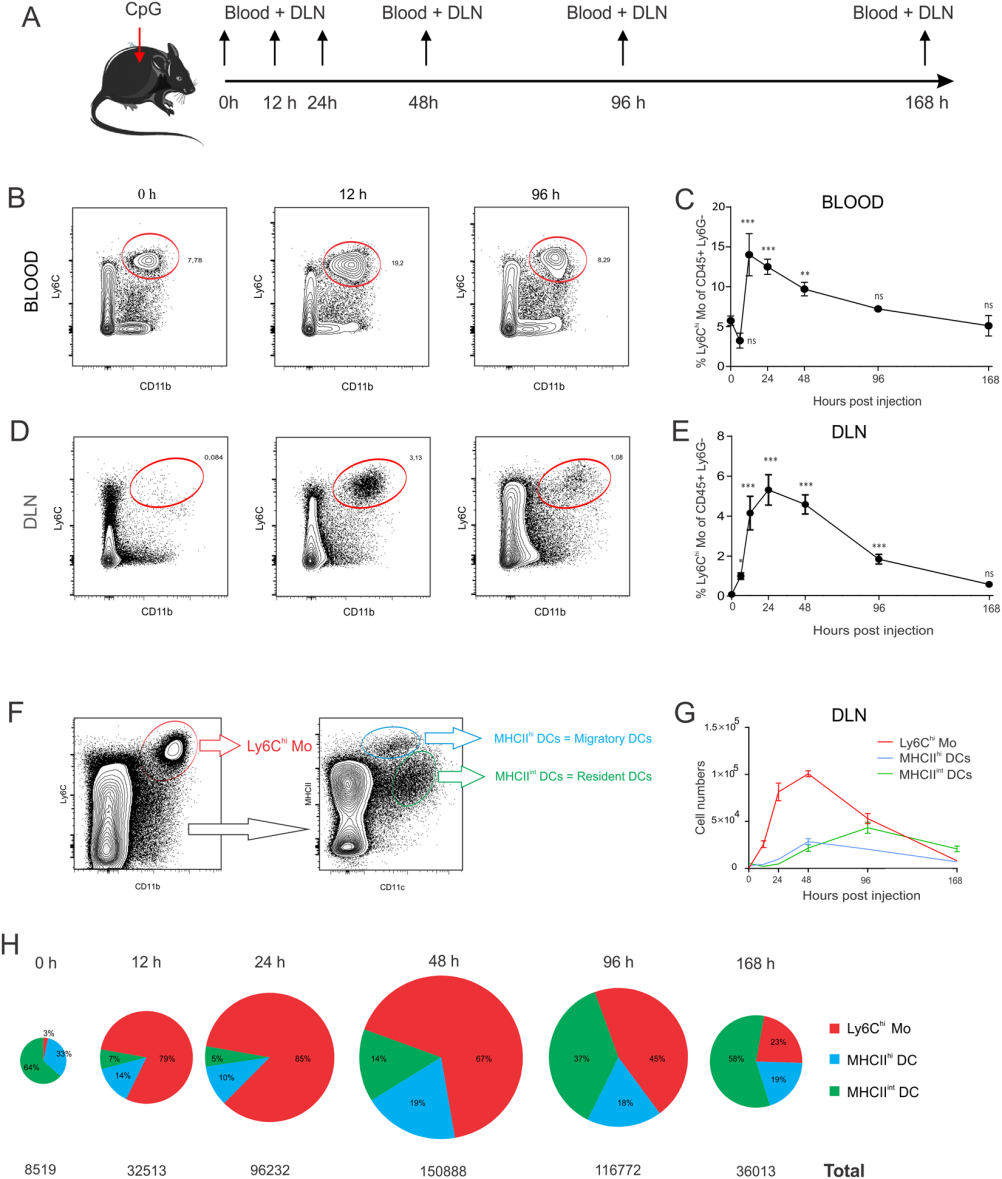
Subcutaneous CpG injection mobilizes Ly6C^hi^ Monocytes and DCs. (**A**) C57Bl/6 mice (n = 4) were injected in the footpad with 20 µl of CpG (100 µg/ml) and blood samples and draining popliteal lymph nodes were analysed at the indicated time intervals by flow cytometry. (**B**,**D**) Flow cytometry plots showing the frequency of Ly6C^hi^ Monocytes in blood (**B**) and draining lymph node (**D**) of CpG injected mice at 0 h, 12 h and 96 h post CpG injection. (**C**,**E**) Graph showing the frequency of Ly6C^hi^ monocytes in blood (**C**) and draining lymph nodes (**E**) of CpG injected mice (as percentage of CD45+ Ly6G− leukocytes) over time. Mean values at each time interval where compared to mean values at steady state (0 h) ***P < 0.001, **p < 0.01, *p < 0.05. (**F**) Gating strategy applied to identify migratory DCs and resident DCs in popliteal lymph nodes. (**G**) Graph showing the absolute numbers of Ly6C^hi^ monocytes, migratory DCs and resident DCs in the draining lymph nodes of OVA/CpG injected lice at the indicated time intervals (n = 4; mean +/− SD). (**H**) Pie charts depicting the relative proportions of Ly6C^hi^ monocytes, migratory DCs and resident DCs in the draining lymph nodes. The area of the circles displayed is relative to the added counts of Ly6C^hi^ monocytes, migratory DCs and resident DCs at the indicated time intervals. Data shown are representative of three independent experiments.

As the major goal of this study was to address the role of Ly6C^hi^ monocytes in the regulation of T cell immunity to CpG adjuvanted vaccines, we first characterized the mobilization of Ly6C^hi^ monocytes to the blood and the vaccine draining lymph nodes at the indicated time intervals post CpG injection (Fig. 1A). Ly6C^hi^ monocytes were identified as live, CD45+ Ly6G− Ly6C^hi^ CD11b^hi^ cells. An overview of the gating strategy is shown in Fig. S1A. The fraction of Ly6C^hi^ monocytes rapidly increased in the blood of CpG injected mice, peaked at around 15% of all CD45+ Ly6G− leukocytes at 12 hours post injection and subsequently declined to baseline over time (Fig. 1B,C). This rapid mobilization of Ly6C^hi^ monocytes to the blood almost coincided with their emergence in the draining lymph node, where Ly6C^hi^ monocytes showed peak percentages between 12 h and 48 h post injection (Fig. 1D,E). Injection of the model antigen ovalbumin (OVA) without CpG did not result in significant expansion of Ly6C^hi^ monocytes in blood or draining lymph nodes (Fig. S1B).

In addition to Ly6C^hi^ monocytes, we also quantified MHCII^hi^ DCs and MHCII^int^ DCs in the draining lymph nodes of CpG injected mice. MHCII^hi^ DCs and MHCII^int^ DCs correspond to respectively migratory DCs and lymph node resident DCs and were identified based on their relative expression of CD11c and MHCII after exclusion of the Ly6C^hi^ monocyte population (Fig. 1F). The absolute cell numbers of Ly6C^hi^ monocytes, migratory DCs and resident DCs in the draining lymph node are shown in the graph in Fig. 1G. The relative proportions of Ly6C^hi^ monocytes, migratory DCs and resident DCs over time are depicted as pie charts (Fig. 1H). The surface area of each pie chart is proportional to the added counts of Ly6C^hi^ monocytes, migratory DCs and resident DCs. At the peak of their recruitment to the draining lymph node, Ly6C^hi^ monocytes were 360 times more abundant when compared to steady state. Migratory DCs and resident DCs also showed a strong expansion, albeit less pronounced and with delayed kinetics when compared to Ly6C^hi^ monocytes. Migratory DCs were about 10-fold increased at their peak of recruitment 48 h post injection. Resident DCs peaked at 96 h post CpG injection, when they showed an 8-fold increase compared to steady state lymph nodes. While Ly6C^hi^ monocytes are 10-fold less abundant than migratory DCs and 20-fold less abundant than resident DCs in steady state, this picture thus promptly inversed after CpG injection, with Ly6C^hi^ monocytes becoming about 8.5-fold more abundant than migratory DCs and 17-fold more abundant than resident DCs at the 24 h time interval.

### Flt3L^−/−^ mice and Ccr2^−/−^ mice show impaired effector T cell responses to CpG adjuvanted vaccines

**Figure 2:**
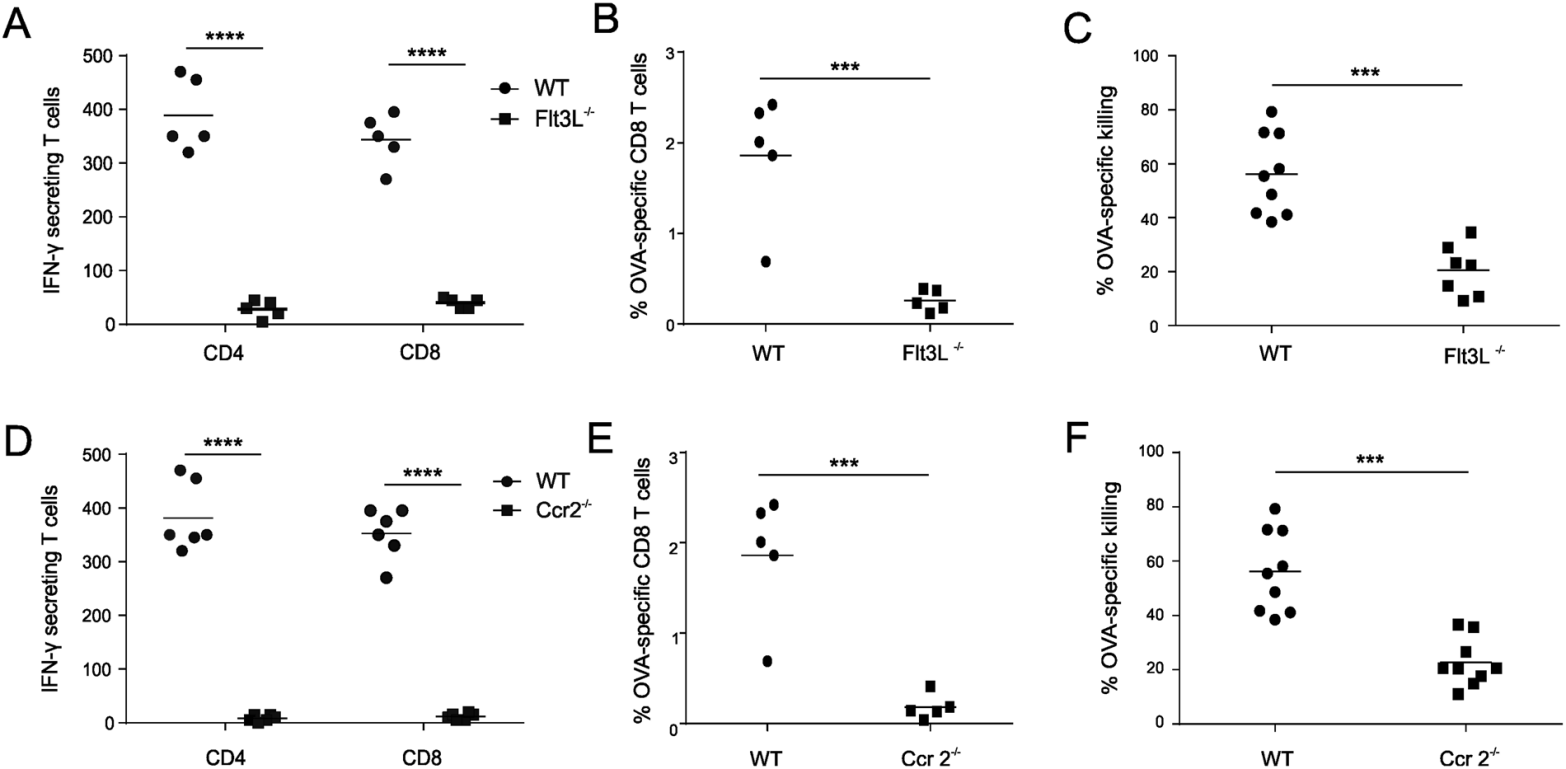
Flt3L^−/−^ mice and Ccr2^−/−^ mice show impaired effector T cell responses. Analysis of the effector T cell response to OVA/CpG vaccination. (**A**–**C**) Comparison of effector T cell responses between WT and Flt3L^−/−^ mice. (**D**–**F**) Comparison of effector T cell responses between WT and Ccr2^−/−^ mice. (**A**,**D**) ELISPOT analysis determining the numbers of IFN-γ secreting T cells (n = 6; mean +/− SD). (**B**,**E**) Flow cytometric quantification of OVA-specific CD8 T cells in blood (n = 5/group; mean +/− SD). (**C**,**F**) OVA-specific cytotoxicity as measured by an *in vivo* killing assay (n = 7/group; mean +/− SD). ****P < 0.0001, ***p < 0.001. Data shown are representative of three independent experiments.

Conventional DC development critically relies on Flt3L^25, 27, 51, 52^, causing mice deficient for Flt3L to display heavily reduced numbers of conventional DCs in skin^30^, lung^32^ and lymphoid organs^53^. To assess the impact of conventional DC deficiency on the effector T cell response, we immunized Flt3L^−/−^ mice with OVA/CpG. In comparison to wild type mice, Flt3L^−/−^ mice showed a dramatic reduction in the numbers of IFN-γ secreting CD4 and CD8 T cells as measured by ELISPOT (Fig. 2A). Flt3L^−/−^ mice also showed less OVA-specific CD8 T cells in blood (Fig. 2B). Functionally, this reduced presence of OVA-specific CD8 T cells translated into a severely hampered cytolytic T cell activity in immunized Flt3L^−/−^ mice as assessed through an *in vivo* killing assay (Fig. 2C).

Using the same set of assays, we next addressed whether monocyte deficiency would also impact the effector T cell response. Although CCR2 is not required for Ly6C^hi^ monocyte development, it is vital for the egression of Ly6C^hi^ monocytes out of the bone marrow thus causing impaired recruitment of Ly6C^hi^ monocytes to sites of inflammation in Ccr2^−/−^ mice. Quantification of Ly6C^hi^ monocytes in the draining lymph nodes confirmed that Ly6C^hi^ monocytes are indeed near absent from the draining lymph nodes of CpG injected Ccr2^−/−^ mice (Fig. S2). Strikingly, also Ccr2^−/−^ mice displayed a dramatic reduction in vaccine elicited effector T cell responses with impaired IFN-γ secretion (Fig. 2D), lower levels of blood OVA-specific CD8 T cells (Fig. 2E) and compromised cytolytic activity (Fig. 2F).

Combined, these studies thus show that generation of effector T cell responses to OVA/CpG necessitates monocytes in addition to conventional DCs, thereby warranting us to further assess monocyte function in dept.

### Ly6C^hi^ monocytes adopt a DC-like phenotype and infiltrate the T cell zone of the draining lymph node

**Figure 3:**
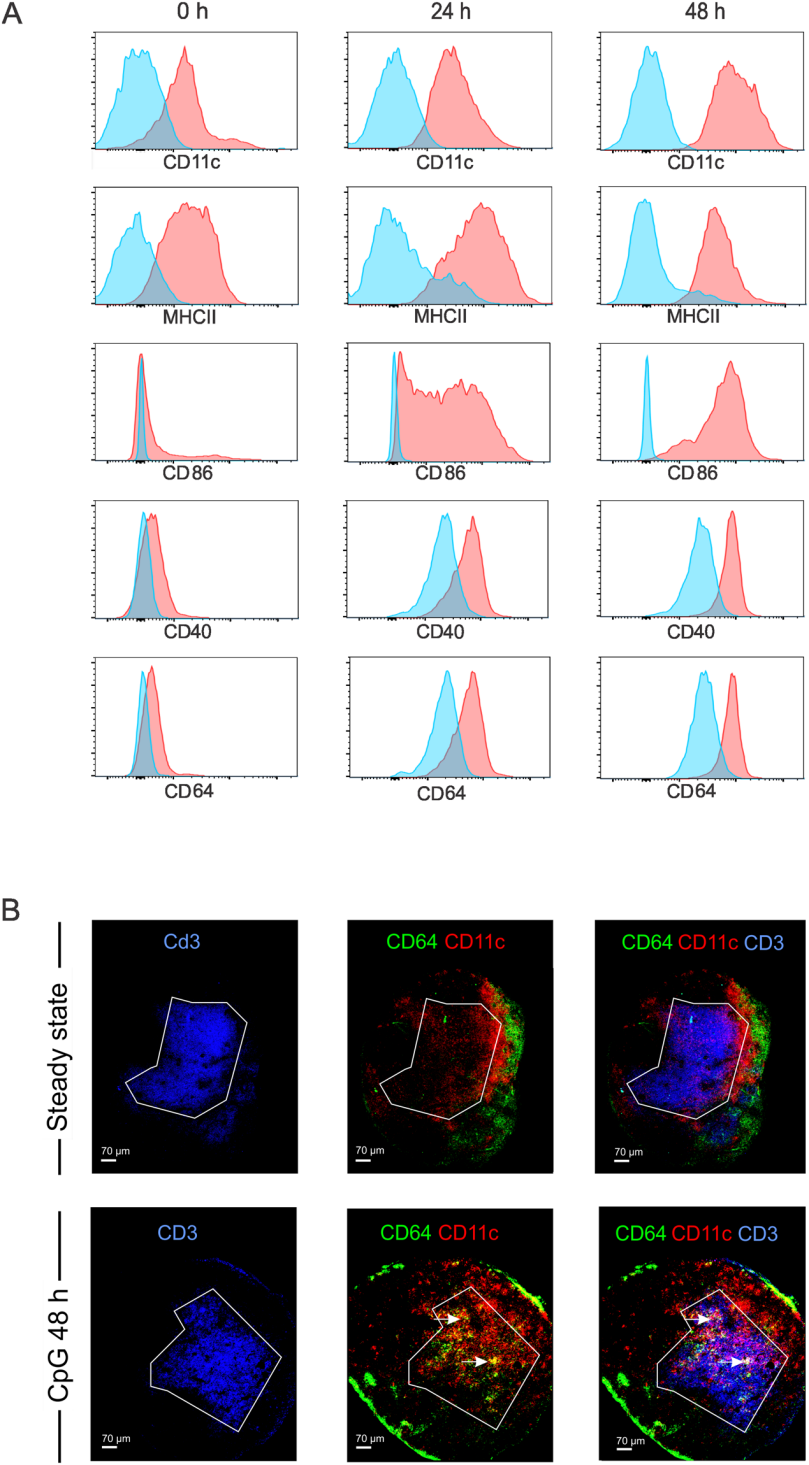
Phenotypic characterization and intranodal distribution of Ly6C^hi^ monocytes. (**A**) Phenotypic characterization of Ly6C^hi^ monocytes in the draining lymph node in response to subcutaneous CpG injection. The levels of CD11c, MHCII, CD86, CD40 and CD64 of ly6C^hi^ monocytes are shown at 0 h, 24 h and 48 h after subcutaneous CpG injection. (**B**) Confocal images of popliteal lymph nodes in steady state and following footpad injection of CpG. Sections were stained for CD3 (blue), CD11c (red) and CD64 (green) to visualize T cells, DCs and Ly6C^hi^ monocytes.

First, we performed a flow cytometric immunophenotyping of Ly6C^hi^ monocytes over time (Fig. 3A). The limited population of Ly6C^hi^ monocytes present in steady state lymph nodes expressed low to intermediate levels of CD11c and MHCII and showed little expression of the co-stimulatory molecules CD86 and CD40. When analyzed at 24 hours and 48 hours post CpG injection, the vast population of freshly recruited Ly6C^hi^ monocytes displayed increased levels of CD11c, MHCII, CD40 and a pronounced upregulation of CD86, suggestive for involvement in T cell priming and activation. CpG recruited Ly6C^hi^ monocytes are thus reminiscent of the inflammatory DCs or monocyte-derived DCs described by others^28, 31, 46, 54–57^. As the high affinity IgG receptor FcyRI (CD64) has recently emerged as a selective marker for monocyte derived DCs^37^, we also analyzed CD64 expression over time. At steady state, the few Ly6C^hi^ monocytes present displayed low levels of CD64. Nonetheless, CpG recruited Ly6C^hi^ monocytes showed a strong upregulation of CD64 upon differentiation into monocyte derived DCs.

Based on the data above, CpG evoked Ly6C^hi^ monocytes appear to acquire the necessary machinery to efficiently interact with T cells. To assess whether Ly6C^hi^ monocytes physically interact with T cells, we cryo-sectioned the draining lymph nodes of mice 48 h after CpG injection, when Ly6C^hi^ monocytes peak and display a CD64^hi^ CD11c^int^ phenotype. Sections were stained for CD3 to identify T cells and for CD11c to identify DCs. Because Ly6C is widely expressed by non-monocyte populations, we used CD64 as marker to stain for monocytes and monocyte derived DCs. Besides monocytes, CD64 expression is restricted to macrophages^24, 58^. In steady state lymph nodes, CD11c+ DCs were visible within the T cell zone whereas CD64+ cells were excluded from the T cell zone and predominantly localized to the subcapsular and medullary sinuses. In addition, these CD64+ cells did not co-stain for CD11c and thereby must correspond to macrophages. CpG injection altered the intranodal distribution of CD64+ cells. Besides being localized in the lymph nodes’ sinuses, large clusters of CD64+ cells now became visible within the T cell zone. These T cell zone infiltrating CD64+ cells also co-stained for CD11c (Fig. 3B, white arrows) and thereby perfectly matched the characteristic CD64+ CD11c^int-hi^ expression pattern we observed on monocyte derived DCs by flow cytometry.

### Ly6C^hi^ monocytes are the dominant antigen containing population in the draining lymph node

**Figure 4:**
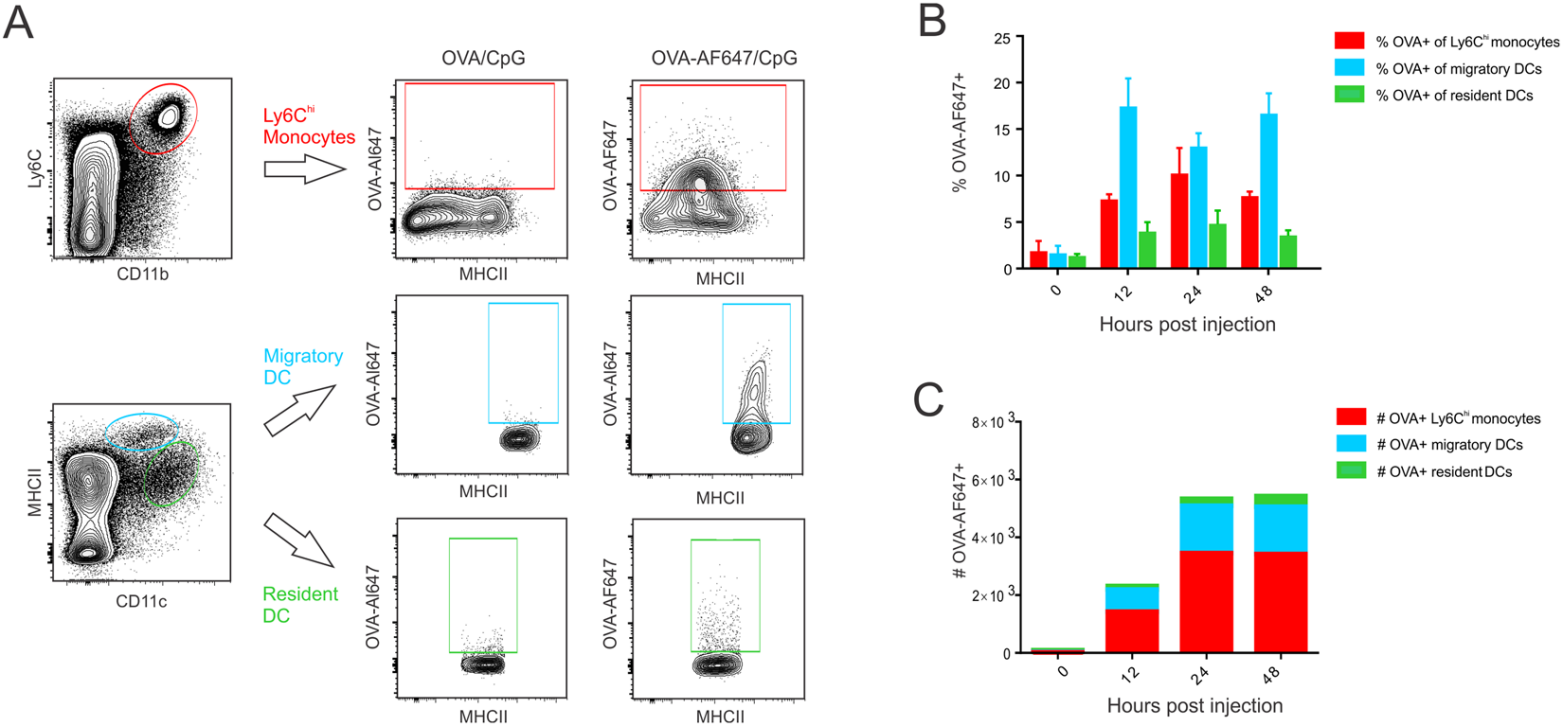
Antigen uptake by monocytes, migratory DCs and resident DCs in lymph nodes, (**A**) Gating strategy applied to identify OVA-AF647+ Ly6C^hi^ monocytes, migratory DCs and resident DCs in the draining lymph nodes of OVA-AF647/CpG injected mice. (**B**,**C**) Graphs showing respectively the percentages (**B**) and absolute numbers (**C**) of Ly6C^hi^ monocytes, migratory DCs and resident DCs that have acquired OVA-AF647 in the draining lymph nodes at the indicated time intervals post OVA-AF647 injection (n = 4). Data are representative of three independent experiments.

Using AlexaFluor647-labeled ovalbumin (OVA-AF647), we monitored antigen uptake by Ly6C^hi^ monocytes, migratory DCs and resident DCs in the draining lymph node. To reliably identify cells as being OVA-AF647+, the same dose of unlabeled OVA mixed with CpG was injected as negative control. An overview of the gating strategy applied is shown in Fig. 4A. In the draining lymph node, migratory DCs showed the largest fraction of OVA-AF647+ cells from 12 h to 48 h post injection (Fig. 4B). Nevertheless, also a significant fraction of Ly6C^hi^ monocytes contained antigen at these time intervals. Resident DCs consistently were the least antigen positive. When looking at the absolute number of antigen positive cells however, Ly6C^hi^ monocytes/monocyte derived DCs were clearly the dominant antigen containing population at 24 h and 48 h post injection (Fig. 4C). At 24 h post CpG injection, when the draining lymph node showed the highest number of OVA-AF647+ cells, OVA containing Ly6C^hi^ monocytes were 3.3 times more abundant compared to OVA containing migratory DCs and almost 20 times more abundant compared to OVA containing resident DCs (n = 5; mean =/− SD). Data shown are representative of three independent experiments.

### Migratory DCs are the major antigen presenting cells in case of CpG adjuvanted protein vaccination

**Figure 5:**
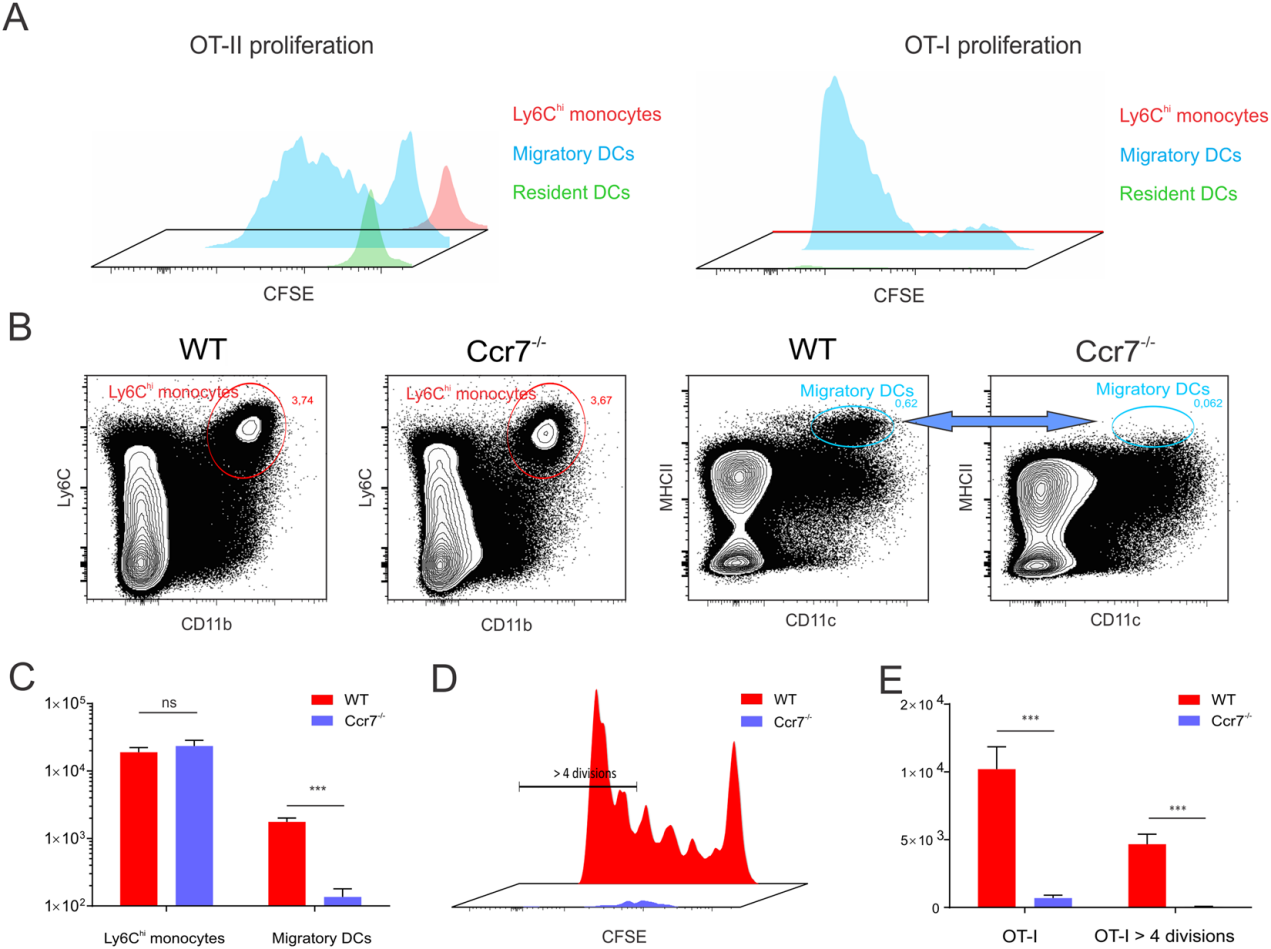
Migratory DCs constitute the major antigen presenting population to CpG adjuvanted protein vaccination. (**A**) Proliferation of CFSE labelled OT-I and OT-II cells following incubation with respectively Ly6C^hi^ monocytes, migratory DCs and resident DCs sorted from the pooled draining lymph nodes of 30 mice injected with OVA/CpG. (n = 5; mean +/− SD). Data shown are representative of two independent sorting experiments. (**B**–**C**) Flow cytometric comparison of Ly6C^hi^ monocyte and migratory DC influx in the draining lymph nodes of CpG injected WT and Ccr7^−/−^ mice. (**B**) Contour plots showing Ly6C^hi^ monocyte and migratory DC frequencies.(**C**) Graph showing the absolute numbers of Ly6C^hi^ monocytes and migratory DCs (n = 5; mean +/− SD). (**D**–**E**) Flow cytometric analysis of OT-I proliferation in the draining lymph nodes of WT and Ccr7^−/−^ mice immunized four days earlier with OVA/CpG. (**D**) Histogram overlay of OT-I proliferation in WT and Ccr7^−/−^ mice. (**E**) Graph quantifying the total numbers of OT-I cells and the number of OT-I cells having undergone over 4 divisions (n = 5; mean +/− SD). Data are representative of three independent experiments.

Because Ly6C^hi^ monocytes acquired antigen, infiltrated the T cell zone of the lymph node and adopted a MHCII^int^ CD11c^int^ DC-like phenotype, we assessed their ability to act as antigen presenting cells. Ly6C^hi^ monocytes as well as migratory DCs and resident DCs were sorted them from the draining lymph nodes of mice immunized 24 hours earlier with OVA/CpG and co-cultured with CFSE labelled OT-I or OT-II cells without further *ex vivo* addition of antigen. As shown in Fig. 5A, Ly6C^hi^ monocytes and resident DCs totally failed to elicit T cell proliferation. In contrast, the migratory DC subset provoked strong OT-I and OT-II proliferation, thereby identifying this DC subset as the prime antigen presenting cell type. Migratory DCs derive from interstitial DCs that have migrated from the skin and carry antigen to the lymph node in a CCR7 dependent fashion^59^. Ccr7^−/−^ mice indeed showed an almost total lack of migratory DCs (MHCII^hi^ DCs) in the draining lymph nodes at 48 h after CpG/OVA injection (Fig. 5B,C) – the time point we showed migratory DCs to peak in wild type mice. Conversely, Ccr7^−/−^ mice showed unaltered Ly6C^hi^ monocyte recruitment, which indicates Ly6C^hi^ monocytes do not require CCR7 for lymph node influx and thus infiltrate the draining lymph node directly from the blood. This combination of dramatically reduced migratory DC influx and unaltered Ly6C^hi^ monocyte recruitment made Ccr7^−/−^ mice ideally suited to address the role of migratory DCs in antigen presentation *in vivo*. As can be appreciated from Fig. 5D,E, proliferation of OVA-specific OT-I T cells was dramatically compromised in OVA/CpG immunized Ccr7^−/−^ mice. These data thus confirm the findings obtained from the *ex vivo* proliferation assay and firmly establish migratory DCs as the major antigen presenting population to CpG adjuvanted protein vaccination.

### Antigen presentation mainly resides within migratory DCs of conventional ontogeny

Recent studies have indicated that Ly6C^hi^ monocytes can strongly downregulate Ly6C expression upon differentiation into monocyte derived DCs in inflamed peripheral tissues^30, 32, 54^. When such monocyte derived DCs subsequently migrate to the draining lymph node, they become Ly6C^lo-int^ CD11b+ CD11c+ MHCII^hi^, which makes them extremely difficult to distinguish from conventional CD11b+ migratory DCs - which are Ly6C− CD11b+ CD11c+ MHCII^hi^ - based on Ly6C expression only. Recently, the combined expression of Ly6C and of CD64 has been documented to enable a more reliable separation of monocyte derived DCs and conventional DCs in murine models of house dust mite allergy and of Alum based immunization^32, 37^.

**Figure 6:**
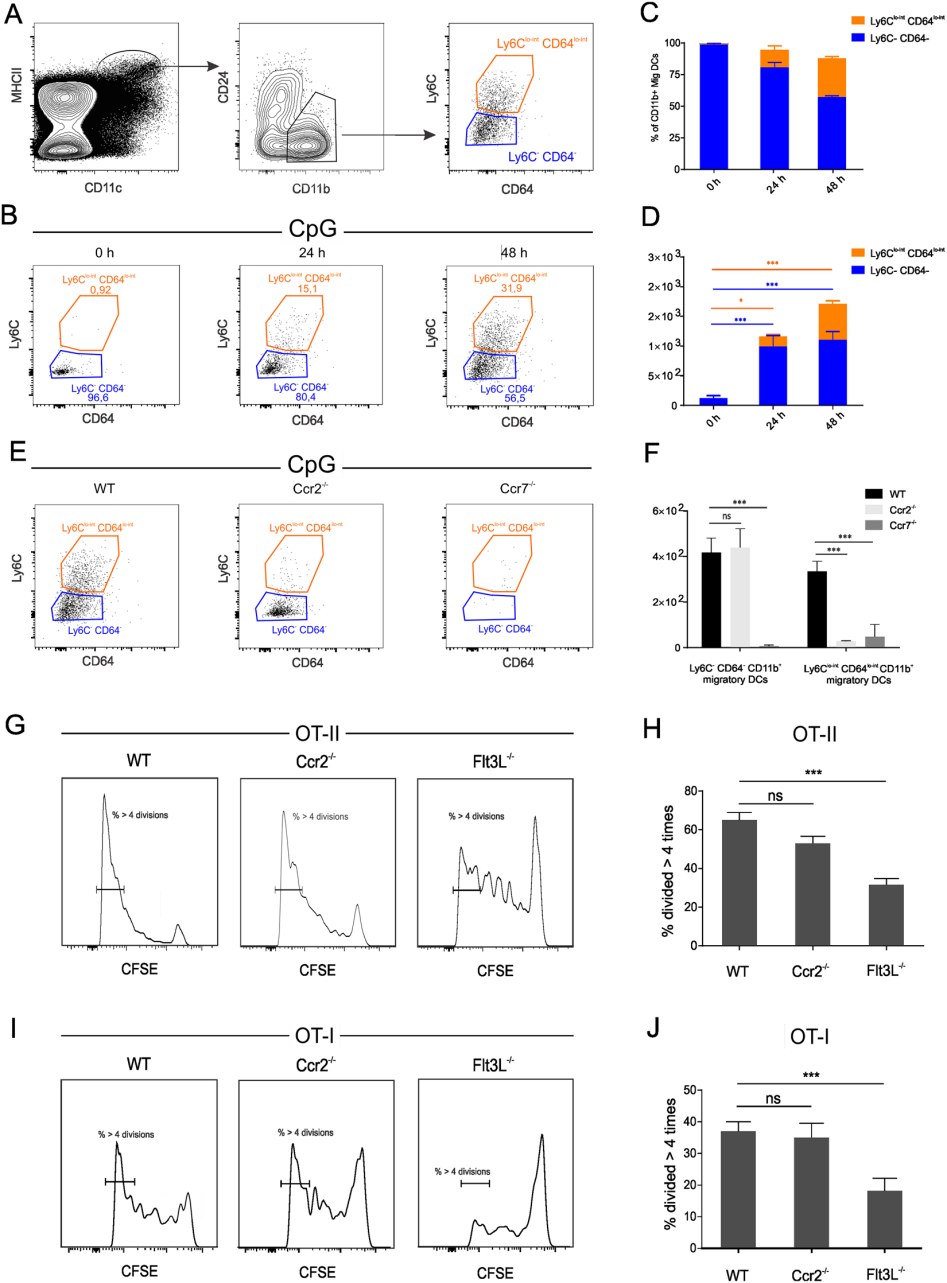
Migratory DCs are of composed of monocyte derived DCs and conventional DCs. (**A**) Gating strategy applied to identify Ly6C^lo-int^ CD64^lo-int^ DCs within the CD11b+ CD24− migratory DC gate. (**B**) Flow cytometry plots of Ly6C and CD64 expression within the CD11b+ CD24− migratory DC at the indicated time intervals post CpG injection. (**C**,**D**) Frequencies and absolute numbers of Ly6C^lo-int^ CD64^lo-init^ CD11b+ CD24− migratory DCs and Ly6C− CD64− CD11b+ CD24− migratory DCs. **(E**,**F)** Dot plots (**E**) and absolute cell counts (**F**) of Ly6C^lo-int^ CD64^lo-int^ CD11b+ CD24− migratory DCs and Ly6C− CD64− CD11b+ CD24− migratory DCs in the draining lymph node of wild type (WT), Ccr2^−/−^ mice and Ccr7^−/−^ mice at 48 h post CpG injection. Data are shown as means +/− SD (n = 5). (**G**,**I**) Histogram plots showing the proliferation of OT-II (**G**) and of OT-I. **(I)** T cells in the draining lymph nodes of OVA/CpG immunized wild type (WT), Ccr2^−/−^ mice and Flt3L^−/−^ mice. (**H**,**J**) Graphs displaying the percentages of OT-II (**H**) and OT-I (**J**) T cells having undergone over 4 divisions in the draining lymph nodes of wild type (WT), Ccr2^−/−^ mice and Flt3L^−/−^ mice (n = 6; means +/− SD). Data are representative of two independent experiments.

To delineate whether cells of monocyte origin contribute to the antigen presenting migratory DC population, we monitored the expression of Ly6C and CD64 on the CD24− CD11b+ MHCII^hi^ migratory DC population before and after OVA/CpG injection. Figure 6A depicts the gating strategy applied, whereas Fig. 6B depicts the expression of Ly6C versus CD64 on CD24− CD11b+ migratory DCs at the indicated time intervals after CpG injection. In steady state lymph nodes, virtually all CD11b+ migratory DCs lacked Ly6C and CD64, in accordance with their described conventional DC nature. Upon CpG injection, a marked increase in the proportion and absolute numbers of CD11b+ migratory DCs that displayed a Ly6C^lo-int^ CD64^lo-int^ phenotype was observed (Fig. 6B–D). To unambiguously mark these Ly6C^lo-int^ CD64^lo-int^ CD11b+ MHCII^hi^ DCs as migratory DCs of monocyte origin, we quantified their numbers in the draining lymph nodes of respectively CpG injected wild type mice, Ccr2^−/−^ mice and Ccr7^−/−^ mice (Fig. 6E,F). In line with a monocyte origin, the Ly6C^lo-int^ CD64^lo-int^ CD11b+ migratory DC population was near absent in Ccr2^−/−^ mice. Conversely, the Ly6C− CD64− migratory DC population was unaffected by CCR2 deficiency, confirming these cells correspond to *bona fide* conventional DCs. Both CD11b+ migratory DC subtypes were strongly affected by CCR7 deficiency, which shows that migratory DCs depend on CCR7 to reach the lymph nodes regardless of a monocyte or conventional DC origin. Very little is known regarding the capacity of monocyte derived DCs to present antigen *in vivo*. To address the relative contributions of monocyte derived migratory DCs and conventional migratory DCs to antigen presentation, we compared the proliferation of adoptively transferred OT-I and OT-II T cells between wild type mice, Flt3L^−/−^ mice and Ccr2^−/−^ mice immunized with OVA/CpG. Within the migratory DC population, Ccr2^−/−^ mice lack monocyte derived DCs while Flt3L^−/−^ mice have a deficit in conventional DCs. Little or no differences in the percentages of OT-II (Fig. 6G,H) or OT-I (Fig. 6I,J) T cells having undergone extensive T cell proliferation were observed between WT and Ccr2^−/−^ mice, arguing against a major role of monocytes and their monocyte derived migratory DC descendants in antigen presentation. Conversely, the deficit in conventional DCs present in Flt3L^−/−^ mice did cause a strongly diminished T cell proliferation in these mice. Although these data do not formally exclude that migratory monocyte DCs can present antigen, they do identify conventional migratory DCs as the most important antigen presenting DC population.

### Ly6C^hi^ monocytes constitute the major source of IL-12 that drives Th1 oriented T cell immunity

**Figure 7:**
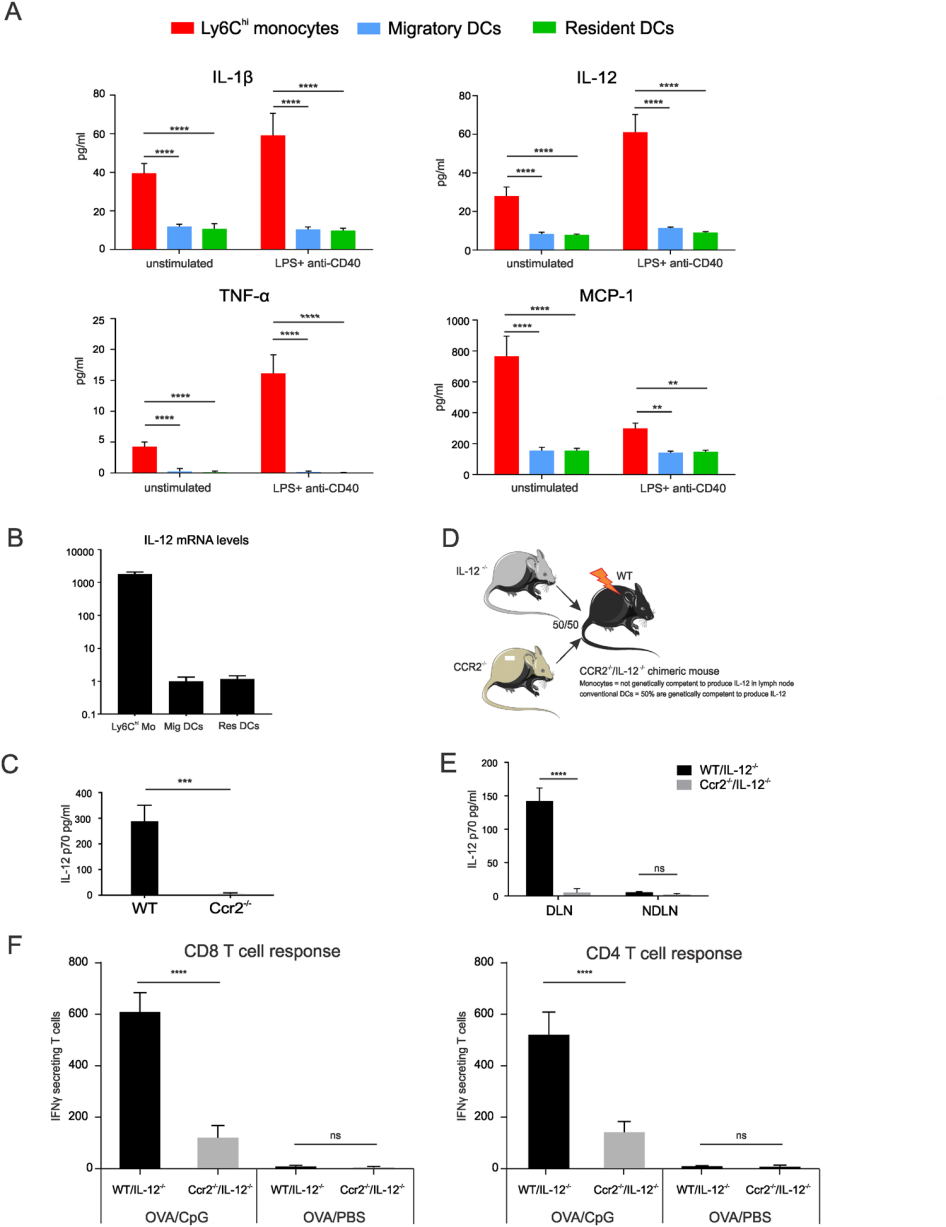
Ly6C^hi^ monocytes constitute the major source of IL-12 that drives IFN-γ T cell responses. (**A**) Graphs showing cytokine production by Ly6C^hi^ monocytes, migratory DCs and resident DCs sorted from the pooled popliteal lymph nodes of mice (n = 40) injected with CpG 24 h earlier. 2 × 10^5^ sorted cells were cultured for 48 h either without further stimulation or stimulated with a low dosis of LPS (100 ng/ml) and anti-CD40 (1 µg/ml). All data points indicate means +/− SD for five replicates. (**B**) Relative IL-12 mRNA transcript levels in Ly6C^hi^ monocytes, migratory DCs and resident DCs sorted from the pooled popliteal lymph nodes of mice (n = 20) injected with CpG 24 h earlier. (**C**) IL-12p70 titers determined in the supernatans of the draining lymph nodes isolated from CpG injected WT and Ccr2^−/−^ mice. Lymph nodes were isolated 24 h post CpG injection and cultured for an additional 48 h *ex vivo* (n = 5; mean +/− SD). (**D**) Schematic overview of the procedure applied to generate mixed IL-12^−/−^/Ccr2^−/−^ chimeric mice. (**E**) IL-12 p70 titers in the supernatans of the draining and non-draining lymph nodes of CpG injected WT/IL-12^−/−^ and Ccr2^−/−^ /IL-12^−/−^ chimeric mice (n = 5; mean +/− SD). (**F**) ELISPOT quantification of the numbers of IFN-γ secreting T cells of WT/IL-12^−/−^ and Ccr2^−/−^ /IL-12^−/−^ chimeric mice immunized with OVA/CpG through a prime booster schedules (n = 5; mean +/− SD). Data shown are a representative of two independent experiments.

Despite monocytes having no major contribution in antigen presentation to naïve T cells, effector T cell responses are dramatically impaired in OVA/CpG immunized Ccr2^−/−^ mice. Consequently, we explored whether Ly6C^hi^ monocytes would be an important source of IL-12, the most prominent signal 3 cytokine driving Th1 oriented immunity. To this end, we sorted Ly6C^hi^ monocytes, migratory DCs and resident DCs from the pooled draining popliteal lymph nodes of mice injected 24 h earlier with OVA/CpG in the footpad. Sorted cells were subsequently cultured either without further stimulation or stimulated with a low dose of LPS and anti-CD40 as previously described by Nakano *et al*.^47^ Strikingly, Ly6C^hi^ monocytes comprised the sole population that secreted detectable amounts of IL-12 - even in the absence of further LPS/CD40 stimulation (Fig. 7A). Ly6C^hi^ monocytes also produced high levels of the inflammatory cytokines IL-1β, TNF-α and IL-6, whereas these cytokines were not detected in the supernatant of cultured migratory DCs and resident DCs. To avoid confounding results due to potential differences in viability between *ex vivo* cultured monocytes and DCs, we determined the relative transcript levels of IL-12 p35 in freshly sorted Ly6C^hi^ monocytes, migratory DCs and resident DCs. In line with the results from the *ex vivo* culture, sorted Ly6C^hi^ monocytes showed far higher transcript levels of IL-12 compared to migratory and resident DC populations (Fig. 7B). This prominent role of Ly6C^hi^ monocytes in IL-12 production was further confirmed by quantification of IL-12 titers in the supernatans of lymph nodes obtained from wild type and Ccr2^−/−^ mice injected with CpG. Consistent with the sorting results, the lack of Ly6C^hi^ monocytes in Ccr2^−/−^ mice virtually abrogated IL-12 production in the draining lymph nodes of CpG injected Ccr2^−/−^ mice (Fig. 7C).

To determine whether monocyte-derived IL-12 is vital for the induction of IFN-γ producing effector T cells, we exploited the selective dependency of Ly6C^hi^ monocytes but not conventional DCs on CCR2 to reach the draining lymph nodes by generating Ccr2^−/−^/IL-12^−/−^ mixed chimeric mice (Fig. 7D). Lethally irradiated wild type mice were reconstituted with respectively a 1:1 bone marrow mixture of wild type mice and IL-12^−/−^ mice or a 1:1 bone marrow mixture of Ccr2^−/−^ mice and IL-12^−/−^ mice. In wild type/IL-12^−/−^ chimeric mice, half of the Ly6C^hi^ monocytes and half of the conventional DCs derive from the wild type background and are thereby genetically competent to produce IL-12. In Ccr2^−/−^ /IL-12^−/−^ chimeric mice, half of the conventional DCs (derived from the Ccr2^−/−^ background) are still genetically competent to produce IL-12 but only monocytes derived from the IL-12^−/−^ background can reach the lymph nodes. Quantification of IL-12 titers in the culture supernatans of the draining lymph nodes of wild type/IL-12^−/−^ mice and of Ccr2^−/−^/IL-12^−/−^ chimeric mice injected with CpG validated that IL-12 production is indeed heavily compromised when monocytes cannot produce IL-12 (Fig. 7E). To address the functional impact of this monocyte derived IL-12 on the effector T cell response, we next immunized wild type/IL-12^−/−^ mice and Ccr2^−/−^/IL-12^−/−^ mice with OVA/CpG and determined the numbers of IFN-γ secreting T cells by ELISPOT. In comparison to wild type/IL-12^−/−^ chimeric mice, Ccr2^−/−^/IL-12^−/−^ chimeric mice showed a 3.7-fold reduction in the number of IFN-γ secreting CD4 T cells and a 5-fold reduction in the number of IFN-γ secreting CD8 T cells. Taken together, these data identify monocytes as the major source of IL-12 and stress the importance of monocyte derived IL-12 for optimal induction of IFN-γ secreting CD8 and CD4 T cells (Fig. 7E).

## Discussion

Due to their capacity to instigate Th1 oriented immunity against co-delivered antigens, CpG oligo’s have emerged as promising vaccine adjuvants. In this study, we have elucidated the main innate immune cells that regulate the effector T cell response to CpG adjuvanted protein vaccines. Our data highlight that the immune-potentiating actions of CpG require the coordinated actions of conventional DCs and monocytes. Antigen presentation in the draining lymph node is predominantly exerted by conventional DCs that have migrated from the inflamed injection site to the draining lymph node. Monocytes contribute little to antigen presentation in this setting, but instead constitute the major source of the signal 3 cytokine IL-12 required to differentiate antigen experienced T cells into Th1 and cytolytic T cells. These data thereby nicely complement a recent study in which monocytes were also shown to support B cell immunity to CpG adjuvanted protein vaccination through inflammatory secretion^49^. More generally, our findings enforce the emerging viewpoint that monocytes orchestrate the characteristics of the T cell response by shaping the local inflammatory environment antigen experienced T cells are exposed to.

Over the past years, monocytes have been implicated in the regulation of T cell immunity either through antigen presentation^34, 46, 60^ or through secretion of cytokines that stimulate effector T cell differentiation^44, 48^. Conversely, an equal number of studies found no involvement of monocytes in antigen presentation^61, 62^ and a recent study by the Steinman lab argued against any involvement of monocytes in shaping T cell immunity upon vaccination with protein antigens adjuvanted with the TLR3 agonist pIC or with the TLR4 agonist MPL^13^. With the role of monocytes emerging as extremely complex and context dependent, we set out to determine whether the vast population of monocytes recruited by CpG is implicated in the strong Th1 polarizing properties of this promising adjuvant. Immunization studies in mice respectively lacking conventional DCs (Flt3L^−/−)^ or monocytes (Ccr2^−/−^) in the vaccine draining lymph node indicated that both monocytes and conventional DCs are required for the Th1 polarized effector T cell response upon CpG based immunization. As Ly6C^hi^ monocytes acquired typical phenotypic features required for antigen presentation – including an upregulated expression of MHCII and of co-stimulatory molecules upon lymph node entry - we hypothesized these monocytes might act as antigen presenting cells. T cell proliferation assays in Ccr2^−/−^ mice and co-cultures between T cells and Ly6C^hi^ monocytes sorted from the draining lymph nodes of CpG injected mice however unmasked the incapacity of Ly6C^hi^ monocytes to present antigen. Instead, sorted migratory DCs were identified to possess potent antigen presenting capacities. The dramatic impairment in T cell proliferation in the lymph nodes of Ccr7^−/−^ mice further strengthened these observations and firmly established migratory DCs as the main antigen presenting cells to OVA/CpG. The crucial role of migratory DCs in antigen presentation is in line with early reports on the antigen presenting capacities of skin derived migratory DCs^18, 59^ and with two recent publications that identified migratory DCs as antigen presenting cells to vaccination with Alum and AS01 as adjuvants^37, 63^. Most monocytes entered the vaccine draining lymph node directly from the blood, displayed a Ly6C^hi^ CD64^hi^ MHCII^int^ phenotype and failed to present antigen. Nevertheless, within the migratory DC population, we also identified a second, far scarcer population of DCs that are of monocytic origin. These monocyte derived migratory DCs expressed lower levels of Ly6C and CD64 compared to their Ly6C^hi^ monocyte counterparts but were still distinguishable from conventional CD11b+ migratory DCs^23, 30, 37^. Similar populations of monocyte derived migratory DCs have been documented in response to Leishmania infection^54^, to house dust mite^32, 38^ and to immunization with Alum or with AS01 as adjuvants^63^. In case of Alum adjuvanted vaccination, sorted monocyte derived migratory DCs were capable of presenting antigen to CD4 and CD8 T cells upon *ex vivo* addition of antigen. Similarly, Plantinga *et al*. demonstrated the capacity of monocyte derived migratory DCs to present antigen to CD4 T cells after house dust mite challenge^32^. Nevertheless, the same study also revealed that monocyte derived DCs are far less efficient in traveling to lymph nodes when compared to their conventional DC counterparts, a feature that restricted their contribution to T cell priming to high doses of house dust mite. Although we were unable to sort sufficient numbers of monocyte derived migratory DCs with adequate purity to directly address their capability to present antigen *ex vivo*, monocyte deficiency has little impact on early T cell proliferation assessed in Ccr2^−/−^ mice, whereas the conventional DC deficit in Flt3L^−/−^ mice results in a marked T cell proliferation reduction. Taken together, these studies thereby demonstrate the necessity of conventional migratory DCs for antigen presentation *in vivo* whilst monocyte derived migratory DCs appear to be largely redundant.

Despite initial proliferation of OVA-specific T cells in Ccr2^−/−^ mice, differentiation of primed T cells into IFN-γ secreting T cells and cytolytic T cells is severely hampered in OVA/CpG immunized Ccr2^−/−^ mice. Two major lines of evidence indicate that these defective effector T cell responses in Ccr2^−/−^ mice are in fact caused by a lack of monocytes that secrete the required IL-12 to drive T cell differentiation. First, when sorted from the draining lymph nodes of CpG injected mice, Ly6C^hi^ monocytes were the most prominent producers of IL-12. Draining lymph node cultures of Ccr2^−/−^ mice and of IL-12^−/−^/Ccr2^−/−^ chimeric mice showed dramatically reduced IL-12 titers. Second, OVA/CpG immunized IL-12^−/−^/Ccr2^−/−^ chimeric mice – where monocytes are present in the lymph node but fail to produce IL-12 - displayed reduced numbers of IFN-γ secreting T cells.

Akin our findings, Nakano *et al*. identified monocyte derived DCs as the sole producers of IL-12 and major instigators of Th1 immunity to immunization with Complete Freund’s Adjuvant (CFA) – which amongst other PRRs triggers TLR9^47^. Monocytes and monocyte derived DCs have also been strongly implicated in the regulation of Th1 immunity to parasite infections that trigger TLR9. Léon *et al*. demonstrated that monocytes differentiate into monocyte derived DCs upon *Leishmania* infection and drive anti-Leishmanial Th1 immune responses through IL-12 production^54^. More recently, monocytes were also shown to provide a robust IL-12 burst in response to *Toxoplasma gondii* infection^64^. Nonetheless, the role of monocytes in these parasitic infections as regulators of Th1 immunity is still contested as other studies identified Batf3 dependent conventional DCs as vital sources of IL-12 to *Leishmania* ^65^ and to *Toxoplasma* infection^66^. A recent study by Shah *et al*. appears to reconcile these apparently opposing findings. In this report, differentiation of CD8 cells into IFN-γ secreting effector T cells upon *Toxoplasma* infection was shown to require the coordinated actions of conventional DCs and monocyte derived DCs, with the former providing a limited early source of IL-12 and the latter supplying a more robust second IL-12 burst that drives effector differentiation of the expanding antigen-experienced cells^64^. In our study, we failed to detect IL-12 in lymph node cultures of sorted migratory and resident Ly6C− DCs – which include the Batf3 dependent DC subset. As Batf3 dependent DCs represent a minor fraction of these sorted migratory and resident Ly6C− DC cultures, our experimental approach might have missed IL-12 production by these DCs. Nonetheless, IL-12 titers of lymph node cultures derived from CpG injected Ccr2^−/−^ and of Ccr2^−/−^/IL-12^−/−^ mice were reduced to baseline, thus firmly establishing monocyte derived DCs as the main source of IL-12. In summary, our findings add to the body of literature now challenging the conservative model of one antigen presenting DCs delivering all three signals that govern T cell immunity. Instead, antigen presenting DCs appear to be assisted by monocytes and potentially other innate immune cells that integrate pathogen derived and environmental cues into the secretion of cytokines that tune the T cell response mainly initiated by conventional DCs. Designing vaccine approaches that harness this astonishing plasticity of monocytes to shape T cell immunity thus represents an interesting avenue to enhance the efficacy of vaccines.

## Methods

### Vaccine formulations and immunizations

OVA was purchased from Worthington. OVA-Al-647 and OVA-Al 488 was purchased from Molecular probes. OVA(257–264) and OVA(323–339) peptide epitopes were purchased from Anaspec Inc. ODN1826 (CpG; TCC ATG ACG TTC CTG ACG TT) is a 20-mer with a nuclease-resistant phosphorothioate backbone and was purchased from Invivogen. ODN1826 is a class B CpG that has potent Th1 skewing properties in mice. Mice were immunized either subcutaneous in the footpad (20 µl volume) or at the tail basis (100 µl volume) with a mixture of OVA (100 µg/ml) and CpG (100 µg/ml).

### Mice

C57BL/6 J mice were purchased from Janvier. OT-I transgenic mice, OT-II transgenic mice and CCR2^−/−^, CCR7^−/−^ and Flt3L^−/−^ mice were bred in house. IL-12 p40^−/−^ mice were a kind gift by dr. E. Muraille (Université Libre de Bruxelles)^67^. All animal experiments were approved by the animal ethical committee of Ghent University. All experiments and procedures were performed in accordance with the relevant guidelines and legislations.

### Kinetics of Ly6C^hi^ Mo and DC recruitment to blood and popliteal lymph nodes

To analyze monocyte and DC recruitment in response to CpG, 20 µl of a 100 µg/ml ODN1864 solution was injected into the footpad of C57BL/6 J mice. At the indicated time intervals post injection, blood samples were collected through the tail vein followed by ACK (Thermo Fisher Scientific) mediated red blood cell lysis. Alternatively, mice were euthanized and the DLNs were dissected. Single cell suspensions of DLN were prepared through incubation with collagenase type IV (Sigma-Aldrich) and passed through a 70 µm cell strainer (BD Falcon).

After treatment with 2.4G2 Ab for 5 min to block the FcR, PBMCs or lymph node cell suspensions were stained with the following anti-mouse Abs: anti-CD45-V450, anti- MHCII-FITC, anti-CD86-PE, anti-CD40- PE, anti-CD64-APC, anti-F4/80-APC, anti-Ly6C-PE-Cy7, anti-CD11b-APC-Cy7 (all BD Biosciences), Ly6G-PerCP-Cy5.5, CCR2-APC (eBioscience), CD64-BV421 (BioLegend) and CD11c-PE-TxR (R & D systems). Death cells were identified by staining with aqua life/dead stain (Invitrogen). Fluorescent events were acquired using an LSRII flow cytometer (BD Biosciences) and analyzed using FlowJo software. After exclusion of dead cells, granulocytes were identified as CD45+ CD11b+ Ly6C+ Ly6G+ cells and Monocytes as CD45+ Ly6C^hi^ CD11b^hi^ Ly6G- cells. Following exclusion of granulocytes and Monocytes in the LN, DCs were subdivided into MHCII^hi^ DCs and MHCII^int^ DCs.

### OT-I and OT-II proliferation assays

The *ex vivo* Ag presentation assays were performed using a protocol adapted from Langlet *et al*. OVA-specific CD8^+^ T and CD4^+^ T cells were isolated from OTI and OTII transgenic mice, respectively, and labeled with 10 µM CFSE. 10^5^ OT-I T cells or 10^5^ OT-II T cells were subsequently co-cultured for 48 hours with respectively 10^4^ sorted Ly6C^hi^ monocytes, MHCII^hi^ DCs or MHCII^int^ DCs obtained from the DLN of mice previously (24 h) immunized with 20 µl of an OVA (100 µg/ml)/CpG (100 µg/ml) mixture. OT-I and OT-II proliferation was determined by the loss of CFSE fluorescence by flow cytometry. Sorts were performed using a FACSAria sorter (BD Biosciences).

*In vivo* antigen presentation assays were performed through adoptive transfer of 2 × 10^5^ CFSE labeled OT-I or OT-II cells to respectively WT C57BL/6 J mice, CCR2^−/−^ mice, CCR7^−/−^ mice and Flt3L^−/−^ mice 48 hours prior to OVA/CpG immunization. Four days post immunization, DLN were dissected, stained with anti-CD3-V450, anti-CD4-PerCP, anti-CD8-PerCP, anti-Vα2 TCR-PE, anti-CD19-APC-Cy7 (all BD Biosciences), PE-labeled SIINFEKL specific dextramers (Immudex) and measured on a LSRII flow cytometer (BD Biosciences).

### Characterization of antigen uptake

To evaluate antigen uptake by Ly6C^hi^ monocytes, MHCII^hi^ and MHCII^int^ DCs in the lymph node, Alexa647-labbeled OVA (100 µg/ml) was injected into the footpad of mice mixed with ODN1864 (100 µg/ml). Popliteal lymph nodes were dissected at the indicated time intervals and single cell suspensions were stained with aqua life/dead, Fc block, CD45-MHCII-FITC, Ly6-PE-Cy7, CD11b-APC-Cy7 (BD-biosciences), CD11c-PE-TxR (R & D systems) and Ly6G-PerCP-Cy5.5 (eBioscience). To determine the OVA-Alexa647 positive monocyte/DC gate, mice were injected with a mixture of unlabeled OVA (100 µg/ml) and ODN1864 (100 µg/ml).

### Generation of chimeric mice

WT C57BL/6 J mice were irradiated with an 8 Gy dose and reconstituted either with a 1:1 mixture of bone marrow from IL-12^−/−^ mice and WT mice or a with 1:1 mixture of bone marrow from IL-12^−/−^ mice and from CCR2^−/−^ mice.

### Quantification of cytokines analysis

To quantify cytokine production in response to CpG injection, DLN were dissected 24 hours post CpG injection and cultured for an additional 24 h *ex vivo* in complete RPMI (Gibco). Cytokine levels of IL-12p70, were determined by Luminex (BioRad) according to the manufacturer’s protocol.

To address cytokine production by sorted Ly6C^hi^ Monocytes, MHCII^hi^ DCs and MHCII^int^ DCs, these cells were sorted from the pooled DLN of 40 mice injected 24 hours earlier with 20 µl of CpG (100 µg/ml). 2 × 10^5^ cells were cultured in a volume of 200 µl either without further stimulation or in the presence of 10 ng/ml LPS (Invivogen) and 1 µg/ml anti-CD40 (BD Biosciences). Cytokine levels, were determined by Luminex (BioRad) according to the manufacturer’s protocol.

### Total RNA preparation and real-time quantitative PCR

The isolation of the RNA was performed with the RNeasy Plus Micro Kit (Qiagen, Hilden, Germany). cDNA was synthesized with an Transcriptor First Strand cDNA synthesis kit (Roche Molecular Systems, Basel, Switzerland). The real-time quantitative PCR was performed on a LightCycler 480 (Roche Molecular Systems, Basel, Switzerland) by using the sensiFAST SYBR No-ROX kit (Bioline, London, United Kingdom). Primers for interleukine 12 p35 (IL12p35) (sense, 5′-AGCCTCCTCCTTGTGGCTA-3′ and antisense, 5′-TGTGCTGGTTTTATCTTTTGTG-3′) and 60 S ribosomal protein L13 a (Rpl13a; reference housekeeping gene for normalization) (sense 5′-CCTGCTGCTCTCAAGGTT-3′ and antisense, 5′-TGGTTGTCACTGCCTGGTACTT-3′) were used.

### Elispot analysis

IFN-γ Elispots were purchase at eBioscience and used according to the manufacturer’s instructions. In brief, 2 × 10^5^ splenocytes of immunized mice were restimulated with respectively 10 µg/ml OVA (257–264) or OVA(323–339) for 24 h on pre-coated Elispot plates.

### *In vivo* killing assay

To quantify the strength of the vaccine evoked cytotoxic T cell response, an *in vivo* killing assay was performed as described earlier^68^. In brief, vaccinated mice were challenged with a 1:1 mixture of peptide pulsed CFSE^lo^ naïve splenocytes (target cells) and CFSE^hi^ unpulsed naïve splenocytes. Four days later, spleen were dissected and the ratio of target versus non-target cells was determined by flow cytometry.

## Electronic supplementary material

Supplementary Information
