# Supplementary material for: Inflammatory monocytes regulate Th1 oriented immunity to CpG adjuvanted protein vaccines through production of IL-12
Source: Sci Rep. 2017 Jul 20;7:5986. doi: 10.1038/s41598-017-06236-6 (PMC5519561; doi:10.1038/s41598-017-06236-6)
Supplement: Supplementary file 1 — Supplementary Information [file 41598_2017_6236_MOESM1_ESM.pdf]

Inflammatory monocytes regulate Th1 oriented immunity to CpG adjuvanted protein vaccines through production of IL-12.

De Koker S[1,3,4*], Van Hoecke L[1,4], De Beuckelaer A[1], Roose K[1,4], Deswarte K[2,5], Willart MA[2,5], Bogaert P[1], Naessens T[1], De Geest BG[6], Saelens X[1,4], Lambrecht BN[2,5], Grooten J[1*]

1. Department of Biomedical Molecular Biology, Ghent University, Ghent, Belgium.
2. VIB Inflammation Research Center, Ghent University, Zwijnaarde, Belgium
3. Department of Biochemistry, Ghent University, Ghent, Belgium
4. VIB Medical Biotechnology Center, Ghent University, Ghent, Belgium
5. Department of Respiratory Medicine, University Hospital Ghent, Ghent, Belgium
6. Department of Pharmaceutics, Ghent University, Ghent, Belgium.

* To whom all correspondence should be addressed

Figure S1

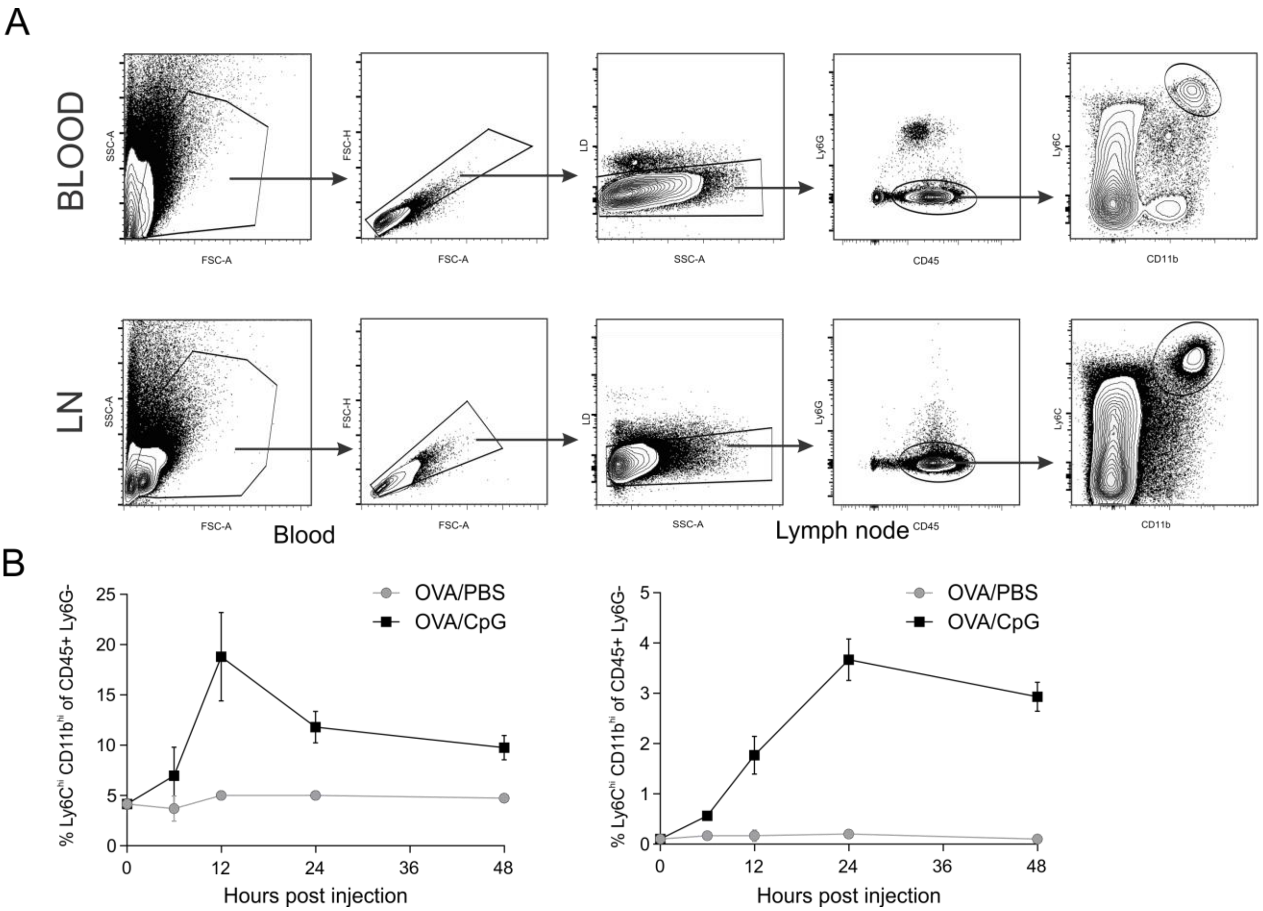

(A) Gating strategy applied to identify Ly6C^{hi} monocytes in blood (upper panel) and lymph nodes (lower panel). **(B)** Graphs showing the percentages of Ly6Chi monocytes in blood (left panel) and lymph nodes (right panel) at the indicated time intervals post OVA/CpG and OVA/PBS injection (n =4; mean +/- SD).

Figure S2

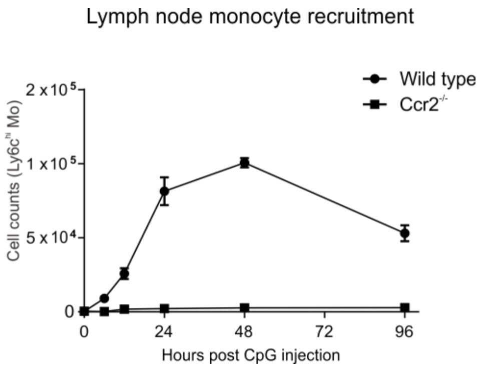

Absolute numbers of Ly6C^{hi} monocytes in the draining lymph node of OVA/CpG injected WT and Ccr2$^{-/-}$ mice at the indicated time intervals (n = 4; mean +/- SD).
